# Died

**Published:** 1863-11

**Authors:** 


DIED.—In Adams, Jefferson Co., N. Y., on Sunday the 18th of October, Dr. Charles E. Brownell, M.D., of this city, aged 23 years. Dr. Brownell was a graduate of the University of Buffalo, and by his energy and ability had established himself in the practice of his profession in a remarkable degree during the brief period allotted him by Providence for the prosecution of professional labor. He was capable, active, and earnest, eminently qualified for distinguished usefulness.

Consumption had marked him for a victim, and though conscious that his work on earth would soon be done, he cheerfully pursued his cherished objects of professional ambition, until exhausted nature could no longer sustain the effort, when, with christian hope, he committed his soul to God.

The profession have sustained the loss of an active, intelligent, and honest physician, while his family, and large circle of friends, mourn the early death of an affectionate husband, a warm friend, and a devoted christian.

